# Co-Delivery of 5-Fluorouracil and Paclitaxel in Mitochondria-Targeted KLA-Modified Liposomes to Improve Triple-Negative Breast Cancer Treatment

**DOI:** 10.3390/ph15070881

**Published:** 2022-07-17

**Authors:** Tianyu Chen, Hui Chen, Yichun Jiang, Qi Yan, Shuling Zheng, Min Wu

**Affiliations:** 1 School of Pharmacy, Chengdu Medical College, No. 783 Xindu Avenue, Xindu District, Chengdu 610500, China; chty1225@163.com (T.C.); jyc13350460277@126.com (Y.J.); shephardyan@163.com (Q.Y.); zsl981013@163.com (S.Z.); 2 School of Healthcare Technology, Chengdu Neusoft University, No. 1 Neusoft Avenue, Dujiangyan District, Chengdu 611830, China; chenhui@nsu.edu.cn

**Keywords:** 5-fluorouracil, paclitaxel, liposomes, mitochondrial targeting, TNBC

## Abstract

In this research, KLA-modified liposomes co-loaded with 5-fluorouracil and paclitaxel (KLA-5-FU/PTX Lps) were developed, and their antitumor activity against triple-negative breast cancer (TNBC) was evaluated. KLA-5-FU/PTX Lps were prepared using the thin-film dispersion method, and their in vitro anticancer efficacy was assessed in human breast cancer cells (MDA-MB-231). An MDA-MB-231 tumor-bearing mouse model was also established to evaluate their antitumor efficacy in vivo. KLA-5-FU/PTX Lps showed enhanced cytotoxicity against MDA-MB-231 cells, improved drug delivery to mitochondria, and induced mitochondria-mediated apoptosis. The modified liposomes also showed favorable antitumor activity in vivo due to their strong ability to target tumors and mitochondria. The liposomes showed no obvious systemic toxicity. Our results suggest that KLA-5-FU/PTX Lps are a promising system with which to target the delivery of antitumor drugs to mitochondria as a treatment for TNBC.

## 1. Introduction

Triple-negative breast cancer (TNBC) is currently considered one of the most threatening malignancies in women [1]. TNBC is characterized by the lack of expression of estrogen receptor (ER), progesterone receptor (PR), and human epidermal growth factor receptor-2 (HER-2), along with an aggressive phenotype and a high tendency towards metastatic progression [2,3]. Due to the lack of specific druggable targets in TNBC, chemotherapy remains the main therapeutic method [4,5], while combination therapy is emerging as an effective strategy to reduce the doses of chemotherapeutic drugs needed and limit their side effects [6].

Paclitaxel (PTX) possesses significant cell-killing activity in a variety of solid tumor cells through its effects on microtubules, cell cycle arrest, and the induction of apoptosis. 5-fluorouracil (5-FU) usually arrests tumor cells at the G_1_-S phase of the cell cycle. PTX and 5-FU are attractive options because of their distinct mechanisms of action, nonoverlapping toxicity, and potential synergy. Many studies have illustrated that PTX combined with 5-FU shows efficacy and tolerability for the treatment of certain solid tumors, particularly primary/metastatic breast carcinoma, drug-refractory ovarian cancers, and advanced/recurrent/metastatic gastric cancer [7,8,9,10,11]. Patients in these studies were treated with different dosages or routes: PTX was administered weekly, biweekly, or triweekly, while 5-FU was administered by bolus injection or continuous infusion with or without other chemotherapeutics in chemotherapy or a neoadjuvant or adjuvant setting [12,13,14,15].

Since PTX and 5-FU are commonly used as first-line chemotherapeutics in patients with metastatic breast cancer [16,17,18,19], their combined administration may serve as a new approach to TNBC treatment. However, successful chemotherapy is limited by the poor solubility and targeting ability of drugs, as well as the emergence of tumor multidrug resistance [20,21]. The distribution of drugs in vivo affects their accumulation at the target site, and the subcellular distribution of drugs will affect their mechanism of action and, in turn, alter their effects [22]. The adoption of various drug nano delivery systems could significantly change the distribution of drugs in vivo and even in subcellular organelles [23]. Thus, a new type of drug delivery system needs to be developed.

**Figure 1:**
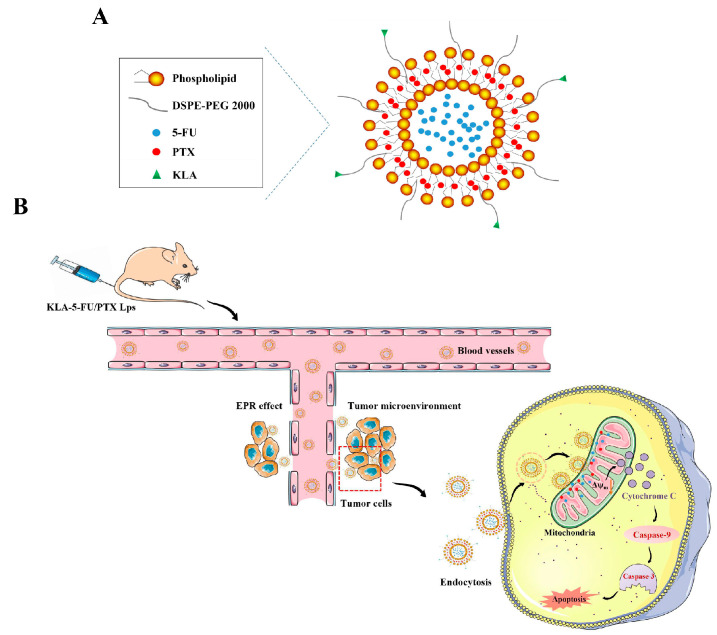
Schematic of (**A**) KLA-5-FU/PTX Lps and (**B**) the mechanism by which they target tumor cells and mitochondria to induce apoptosis. KLA, d-[KLAKLAK]_2_ peptide; 5-FU, 5-fluorouracil; PTX, paclitaxel.

Liposomes (Lps) are lipid-based nanoparticles that can carry both hydrophilic and hydrophobic drugs to achieve a synergistic effect [24]; they can target tumors through the enhanced permeability and retention (EPR) effect [25,26]. In recent years, the targeted delivery of chemotherapeutic drugs to organelles within tumor cells using liposome-based delivery systems has been shown to induce tumor cell death [27]. In particular, targeting drugs to mitochondria in tumor cells can enhance drug efficacy and specificity [28,29] since the mitochondrion is an important subcellular organelle responsible for energy provision and cell apoptosis and is involved in multiple aspects of tumorigenesis and tumor progression [30,31]. For rapidly proliferating TNBC cells, the mitochondrion is an appropriate target for chemotherapy. Targeting PTX and 5-FU to the mitochondria may strengthen their ability to induce apoptosis from their original effects of stabilizing microtubules and interfering with the biosynthesis of DNA and RNA, respectively. However, Lps require further surface functionalization to improve their ability to target mitochondria [32,33].

d-[KLAKLAK]_2_ (hereafter, KLA, Beijing SciLight Biotechnology, Beijing, China) is a non-toxic, positively charged, proapoptotic peptide that can specifically target mitochondria and disrupt their membranes [34,35,36]. Therefore, in this study, we prepared KLA-modified Lps that could deliver and accumulate chemotherapeutic drugs in tumor cell mitochondria, rapidly inducing apoptosis [37]. The prepared Lps were co-loaded with 5-FU and PTX, which showed a good synergistic effect at a fixed dose ratio (Figure 1), and their properties and antitumor activity against TNBC cells were evaluated in vitro and in vivo. The efficiency and improvement of the mitochondrion targeting of KLA-5-FU/PTX Lps were compared with those of free drugs and Lps without mitochondrial-targeting modification.

## 2. Results

### 2.1. Synthesis of DSPE-PEG-KLA

**Figure 2:**
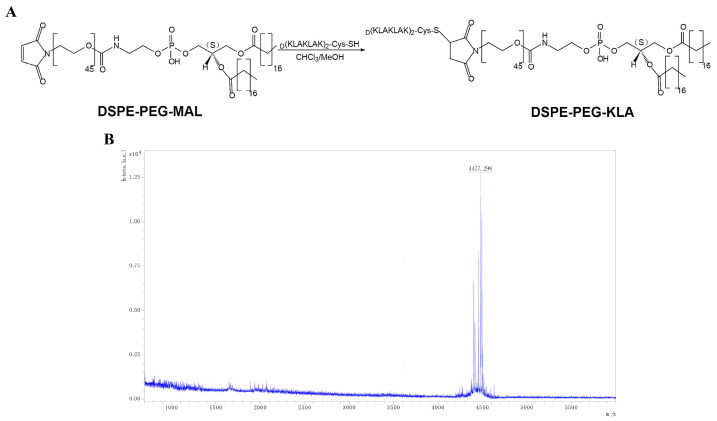
(**A**) Synthesis of DSPE-PEG-KLA and (**B**) its MALDI-TOF-MS spectrum. DSPE-PEG, distearoyl phosphatidyl ethanolamine-polyethylene glycol; KLA, d-[KLAKLAK]_2_ peptide.

DSPE-PEG-KLA was synthesized by conjugating DSPE-PEG-MAL to the cysteine residue of the KLA peptide (Figure 2A). The yield of the final product was 56%. Subsequently, the molecular weight of DSPE-PEG-KLA was determined by MALDI-TOF-MS to be 4477.298 m/z (Figure 2B), consistent with the predicted value.

### 2.2. Preparation and Characterization of KLA-5-FU/PTX Lps

**Figure 3:**
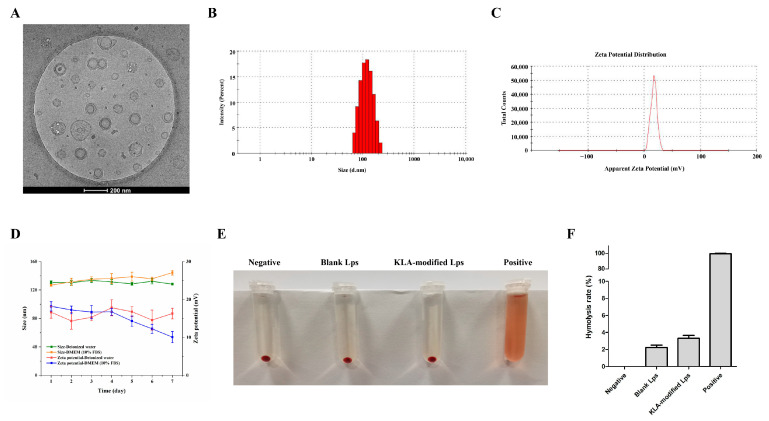
(**A**) Cryo-transmission electron microscopy image, (**B**) size distribution, (**C**) zeta potential, and (**D**) stability of KLA-5-FU/PTX liposomes. The image (**E**) and quantitative analysis (**F**) of hemolysis experiments of blank Lps and KLA-modified Lps. 5-FU, 5-fluorouracil; PTX, paclitaxel.

KLA-5-FU/PTX Lps prepared using the thin-film dispersion method were larger than blank Lps and 5-FU/PTX Lps. The polydispersity index (PDI) of KLA-5-FU/PTX Lps was 0.238 ± 0.011. Moreover, their zeta potential was positive compared to the negative values for blank Lps and 5-FU/PTX Lps, indicating their successful surface modification with the positively charged KLA peptide (Table 1). The entrapment and drug loading efficiencies of 5-FU in KLA-5-FU/PTX Lps were 82.81 ± 2.16% and 7.99 ± 1.36%, respectively, while the respective values for PTX were 80.47 ± 2.39% and 6.53 ± 1.03%. In addition, the Lps showed a smooth, spherical shape (Figure 3A–C).

### 2.3. Stability of KLA-5-FU/PTX Lps

As shown in Figure 3D, in deionized water, KLA-5-FU/PTX Lps remained stable at 4 °C for at least 7 days without significant changes in size or zeta potential. In DMEM containing 10% FBS, due to KLA-5-FU/PTX Lps possessing a positive surface charge, enabling it to bind with negatively charged proteins, the particle size increased by about 20 nm, and the zeta potential decreased by about 10 mV within 7 days. However, overall, the KLA-5-FU/PTX Lps remained stable and positively charged in simulated in vivo conditions. In addition, no significant changes were observed in either physical appearance or particle aggregation in both media, suggesting that the prepared Lps were sufficiently stable for use in further experiments.

### 2.4. Hemolysis Assay of KLA-Modified Lps

As shown in Figure 3E, blank Lps and blank KLA-modified Lps did not cause significant hemolysis. All the hemolytic activity of Lps was less than 4% (Figure 3F). These results indicated that KLA-modified Lps have good biosafety and biocompatibility.

### 2.5. Release of 5-FU and PTX from KLA-5-FU/PTX Lps In Vitro

**Figure 4:**
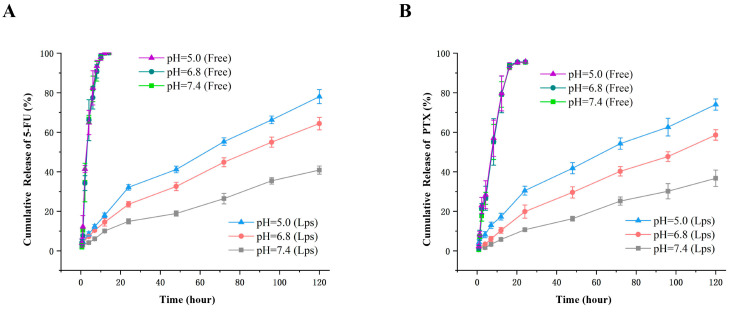
The release profile of free 5-FU (**A**), free PTX (**B**), and KLA-5-FU/PTX Lps from release medium at different pH values. All experiments were performed in triplicate. 5-FU, 5-fluorouracil; PTX, paclitaxel; KLA-5-FU/PTX Lps, KLA-modified liposomes co-loaded with 5-FU and PTX.

The release profiles of 5-FU and PTX from KLA-5-FU/PTX Lps were measured in vitro under different pH conditions. As shown in Figure 4, over time, both 5-FU and PTX in KLA-5-FU/PTX Lps were released much more slowly than the free drug. At 8 h, the cumulative release of the free drug (5-FU) was about 90%, but it was about 10% in the Lps (PTX 60%, 10%). This suggests that, in comparison with free drugs, KLA-5-FU/PTX Lps have sustained-release properties. In addition, the release of the two drugs gradually increased as the pH of the medium decreased, and the drug release increased over time, suggesting that KLA-5-FU/PTX Lps can sustainably deliver both drugs in the acidic tumor microenvironment.

### 2.6. Synergistic Effect of 5-FU and PTX

**Figure 5:**
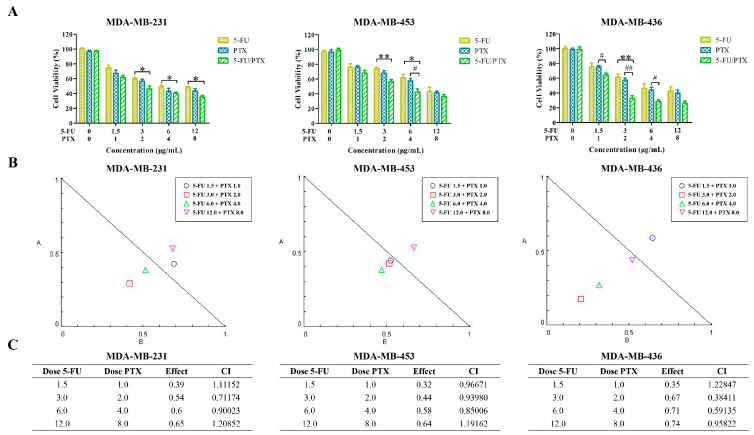
Evaluation of the effect of 5-FU and PTX combinations in MDA-MB-231, MDA-MB-453, and MDA-MB-436 cells. (**A**) MDA-MB-231, MDA-MB-453, and MDA-MB-436 cell viability after treatment with different combinations of 5-FU and PTX. * *p* < 0.05, ** *p* < 0.01; ^#^ *p* < 0.05, ^##^ *p* < 0.01. (**B**) Isobologram plots for combination treatments of 5-FU and PTX in MDA-MB-231, MDA-MB-453, and MDA-MB-436 cells. Lower left of the hypotenuse, synergism; on the hypotenuse, additive effect; upper right, antagonism. (**C**) CI values for MDA-MB-231, MDA-MB-453, and MDA-MB-436 cells. 5-FU, 5-fluorouracil; PTX, paclitaxel; 5-FU/PTX, mechanically mixed free 5-FU and free PTX; CI, combination index.

The synergistic effect of 5-FU and PTX was evaluated in MDA-MB-231, MDA-MB-453, and MDA-MB-436 cells. In three TNBC cell lines, the combination of 5-FU and PTX reduced cell viability more than either drug on its own (Figure 5A). The analysis of the CI values showed that in three TNBC cell lines, most combinations of 5-FU and PTX exhibited synergistic effects, which fell on the lower left of the isobologram (Figure 5B,C). Indeed, combinations with doses of 3 µg/mL 5-FU and 2 µg/mL PTX showed a potent synergistic effect, as suggested by the CI values (0.71174 for MDA-MB-231, 0.93980 for MDA-MB-453, and 0.38411 for MDA-MB-436). Therefore, we chose this concentration of the combination of the two drugs for subsequent experiments.

### 2.7. In Vitro Cytotoxicity

**Figure 6:**
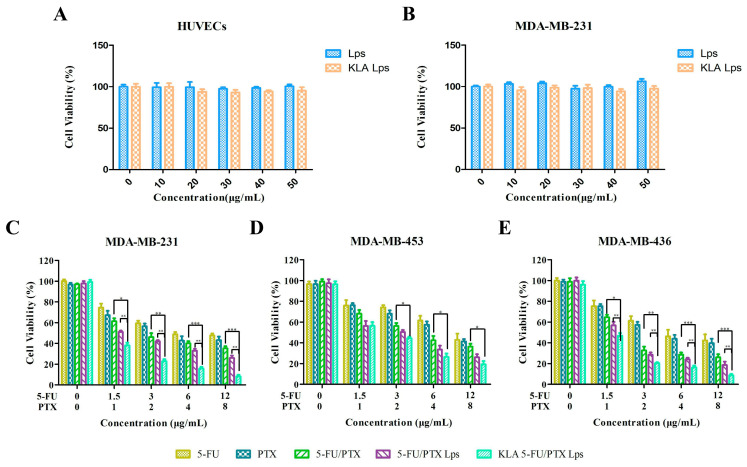
(**A**,**B**) In vitro cytotoxicity of blank Lps and blank KLA Lps against (**A**) human umbilical vein endothelial cells (HUVECs) and (**B**) MDA-MB-231 cancer cells after treatment for 48 h. (**C**–**E**) In vitro cytotoxicity of 5-FU, PTX, 5-FU/PTX, 5-FU/PTX Lps, and KLA-5-FU/PTX Lps against (**C**) MDA-MB-231, (**D**) MDA-MB-453, and (**E**) MDA-MB-436 cancer cells after treatment for 24 h. * *p* < 0.05, ** *p* < 0.01, *** *p* < 0.001. 5-FU, 5-fluorouracil; PTX, paclitaxel; 5-FU/PTX, mechanically mixed free 5-FU and free PTX; 5-FU/PTX Lps, unmodified liposomes co-loaded with 5-FU and PTX; KLA-5-FU/PTX Lps, KLA-modified liposomes co-loaded with 5-FU and PTX.

To evaluate the biocompatibility of KLA-5-FU/PTX Lps, we determined the cytotoxicity of various concentrations of blank Lps and blank KLA-modified Lps against HUVECs and MDA-MB-231 cells. Neither formulation significantly affected cell proliferation at concentrations up to 50 µg/mL (Figure 6A,B), suggesting that KLA-modified Lps may serve as safe, biocompatible carriers of chemotherapeutic drugs.

The cytotoxicity of various formulations was also examined against MDA-MB-231, MDA-MB-453, and MDA-MB-436 cells (Figure 6C–E). Table 2 show the IC_50_ values of different drug groups against different TNBC cells; it can be seen that 5-FU/PTX Lps and KLA-5-FU/PTX Lps exhibited lower IC_50_ values. These results suggest that compared to free drugs, drug-loaded Lps showed stronger cytotoxicity and achieved a good inhibitory effect against the three TNBC cell lines. Among these, KLA-5-FU/PTX Lps showed the best inhibitory effect, indicating that KLA-modified Lps can effectively inhibit TNBC cells and induce a synergistic effect against both drugs.

### 2.8. Lps Uptake by Cells

**Figure 7:**
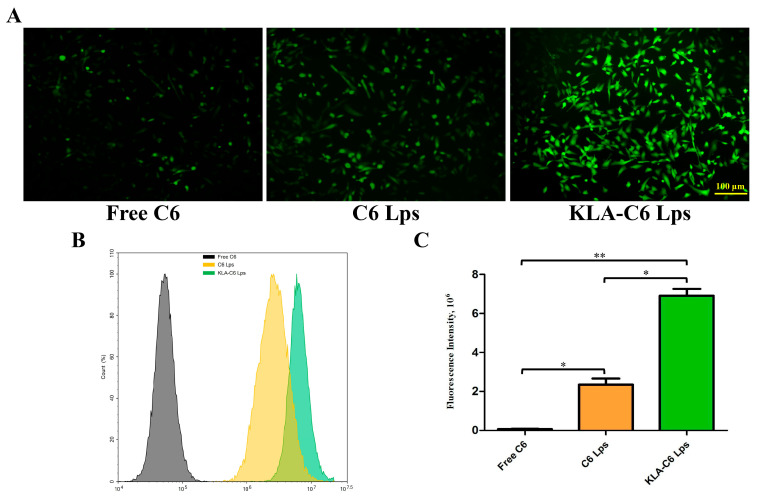
(**A**) Cellular uptake images of MDA-MB-231 cells after treatment with free C6, C6 Lps, and KLA-C6 Lps for 4 h. (**B**) Cellular uptake of C6, C6 Lps, and KLA-C6 Lps detected by flow cytometry for 4 h. (**C**) Semiquantitative analysis of the intracellular uptake of C6, C6 Lps, and KLA-C6 Lps by flow cytometry. * *p* < 0.05, ** *p* < 0.01. C6, coumarin-6; C6 Lps, unmodified C6-loaded liposomes; KLA-C6 Lps, KLA-modified C6-loaded liposomes.

The uptake of free C6, C6 Lps, and KLA-C6 Lps by MDA-MB-231 cells was examined using inverted fluorescence microscopy and flow cytometry. After incubation for 4 h, a stronger green fluorescence was observed in the cytoplasmic region of KLA-C6 Lps-treated cells than in the cytoplasm of cells treated with free C6 or C6 Lps (Figure 7A). The semiquantitative analysis also demonstrated similar results (Figure 7B,C). These results suggest that modification with the KLA peptide promotes the uptake of drugs into tumor cells. This may be related to the lysine moiety of the KLA peptide, which interacts with tumor cell membranes via electrostatic interactions and hydrogen bonding, favoring uptake [38,39].

### 2.9. Apoptosis

**Figure 8:**
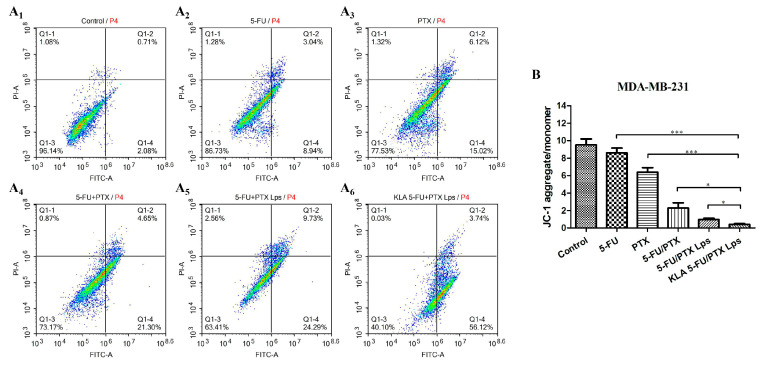
(**A**) Apoptosis of MDA-MB-231 cells treated for 12 h with saline (control), 5-FU, PTX, 5-FU+PTX, 5-FU+PTX Lps, or KLA-5-FU+PTX Lps, as determined by flow cytometry. (**B**) Effect of different formulations on the mitochondrial membrane potential of MDA-MB-231 cells. * *p* < 0.05, *** *p* < 0.001. 5-FU, 5-fluorouracil; PTX, paclitaxel; 5-FU/PTX, mechanically mixed free 5-FU and free PTX; 5-FU/PTX Lps, unmodified liposomes co-loaded with 5-FU and PTX; KLA-5-FU/PTX Lps, KLA-modified liposomes co-loaded with 5-FU and PTX. PI-A, propidium iodide channel; FITC-A, FITC channel.

The apoptosis of MDA-MB-231 cells treated with different formulations was examined using flow cytometry. Among the examined preparations, KLA-5-FU/PTX Lps showed the highest apoptosis rate (59.86%), followed by 5-FU/PTX Lps (34.02%), 5-FU/PTX (25.95%), free PTX (21.14%), and free 5-FU (11.98%) (Figure 8A_1_–A_6_). These results suggest that KLA-modified Lps can efficiently induce apoptosis and enhance the antitumor efficacy of 5-FU and PTX.

### 2.10. Mitochondrial Membrane Potential

Changes in the mitochondrial membrane potential indicate altered membrane permeability, leading to the release of proapoptotic proteins into the cytoplasm and, consequently, to programmed cell death [40]. Here, we found that the JC-1 aggregate/monomer ratio in MDA-MB-231 cells was significantly lower after treatment with KLA-5-FU/PTX Lps than after treatment with the other formulations (Figure 8B). These results indicate that KLA-modified Lps can severely damage mitochondrial function, thereby contributing to cell apoptosis and necrosis.

### 2.11. Mitochondrial Targeting Ability of Lps

**Figure 9:**
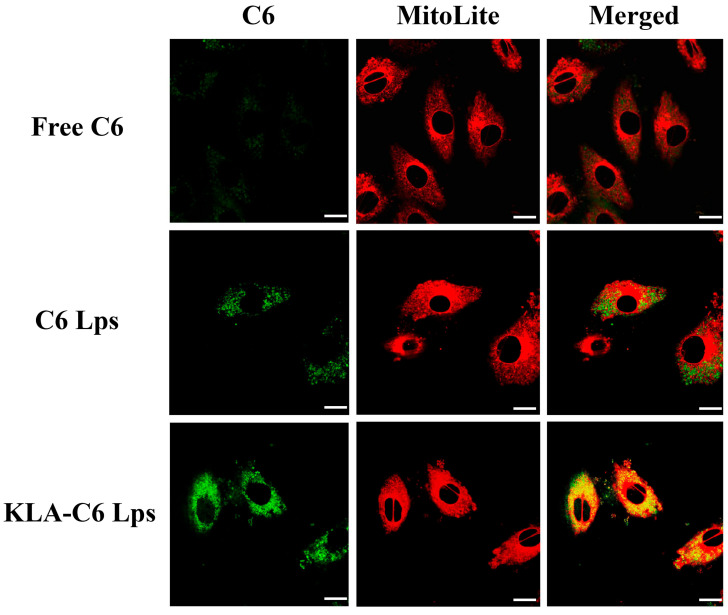
Mitochondrial targeting ability of different formulations, as determined by CLSM. Green fluorescence indicates C6 Lps; red fluorescence, mitochondria; and yellow fluorescence, co-localization of C6 Lps and mitochondria. Scale bar, 10 µm. C6, coumarin-6; C6 Lps, unmodified C6-loaded liposomes; KLA-C6 Lps, KLA-modified C6-loaded liposomes.

To determine whether the antitumor effect of KLA-5-FU/PTX Lps was enhanced via mitochondria-mediated apoptosis, the mitochondrial targeting ability of different formulations was examined using CLSM. As shown in Figure 9, stronger yellow fluorescence was observed in cells treated with KLA-C6 Lps, while a poor co-localization was observed in the free C6 or C6 Lps group, indicating that KLA-modified Lps can successfully target mitochondria and have an affinity for mitochondria in MDA-MB-231 cells.

### 2.12. Caspase-3 Protein Expression

**Figure 10:**
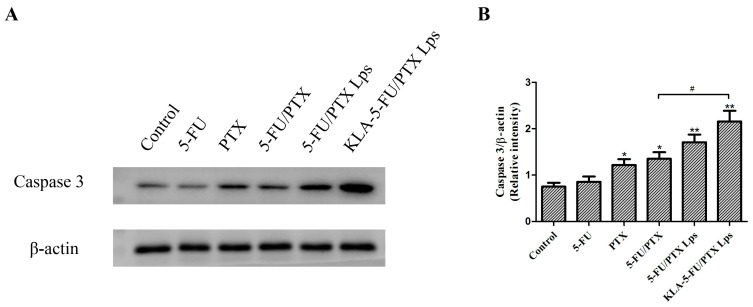
(**A**) Western blots of caspase-3. (**B**) Relative levels of caspase-3, normalized to levels of β-actin. * *p* < 0.05, ** *p* < 0.01 vs. control, ^#^ *p* < 0.05. 5-FU, 5-fluorouracil; PTX, paclitaxel; 5-FU/PTX, mechanically mixed free 5-FU and free PTX; 5-FU/PTX Lps, unmodified liposomes co-loaded with 5-FU and PTX; KLA-5-FU/PTX Lps, KLA-modified liposomes co-loaded with 5-FU and PTX.

Caspase-3 expression in tumor cells has been associated with apoptosis induced by different liposome formulations [41]. Consistent with these results, we found that treatment with 5-FU/PTX, 5-FU/PTX Lps, or KLA-5-FU/PTX Lps significantly increased the caspase-3 expression in MDA-MB-231 cells (Figure 10). In fact, KLA-5-FU/PTX Lps led to significantly higher caspase-3 expression than 5-FU/PTX, confirming that KLA modification can enhance the ability of 5-FU and PTX to induce apoptosis in TNBC cells via mitochondria-mediated apoptosis.

### 2.13. In Vivo Antitumor Activity

**Figure 11:**
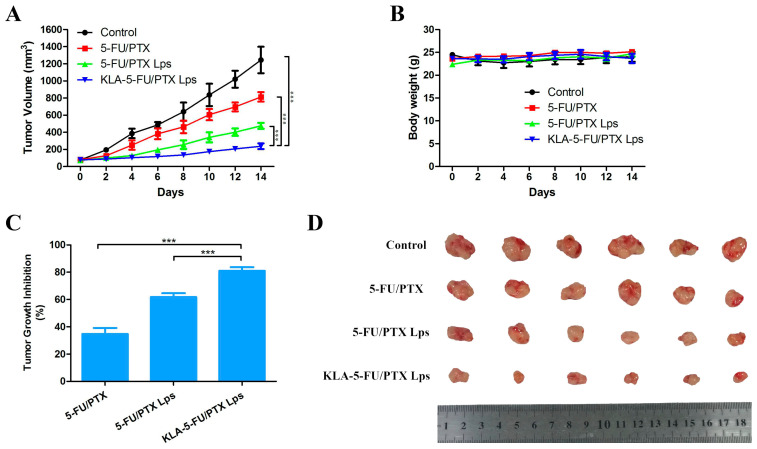
(**A**) Tumor volume, (**B**) body weight, and (**C**) tumor growth inhibition in BALB/c Nude mice bearing MDA-MB-231 tumor grafts and treated with different formulations. (**D**) Tumor tissues collected from each treatment group at 14 days post-administration. *** *p* < 0.001. 5-FU, 5-fluorouracil; PTX, paclitaxel; 5-FU/PTX, mechanically mixed free 5-FU and free PTX; 5-FU/PTX Lps, unmodified liposomes co-loaded with 5-FU and PTX; KLA-5-FU/PTX Lps, KLA-modified liposomes co-loaded with 5-FU and PTX.

The antitumor activity of KLA-5-FU/PTX Lps compared to other formulations was also evaluated in BALB/c Nude mice bearing MDA-MB-231 tumor grafts. Tumor volume rapidly increased in the control groups, reaching a maximum of ~1200 mm^3^ on day 14 (Figure 11A,D). In contrast, KLA-5-FU/PTX Lps successfully inhibited tumor growth by 81% compared to only 34.69% for 5-FU/PTX or 61.8% for 5-FU/PTX Lps (Figure 11A,C). None of the formulations caused obvious weight loss (Figure 11B). These results suggest that KLA-modified Lps are an efficient, safe drug delivery system.

### 2.14. In Vivo Toxicity

**Figure 12:**
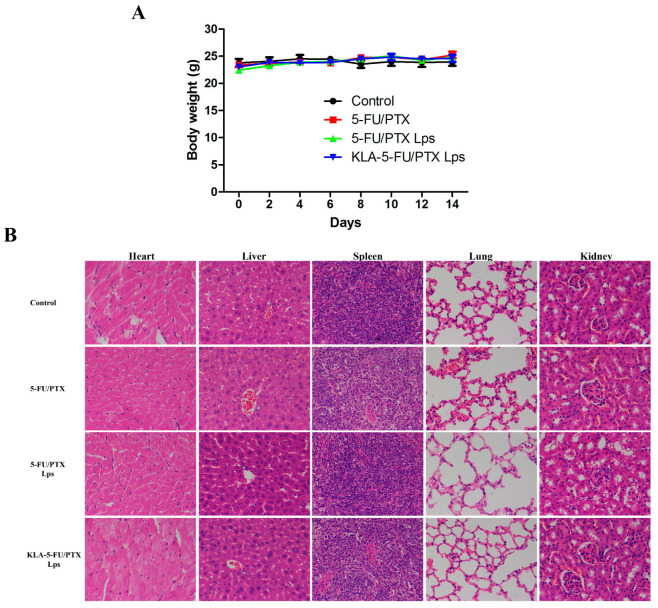
(**A**) Body weight of tumor-free mice treated with different formulations. (**B**) Histopathological examination of the heart, liver, spleen, lung, and kidney of tumor-free mice treated with different formulations. Original magnification, 400×. 5-FU, 5-fluorouracil; PTX, paclitaxel; 5-FU/PTX, mechanically mixed free 5-FU and free PTX; 5-FU/PTX Lps, unmodified liposomes co-loaded with 5-FU and PTX; KLA-5-FU/PTX Lps, KLA-modified liposomes co-loaded with 5-FU and PTX.

The evaluation of the toxicity of the developed formulations in tumor-free mice revealed no significant weight loss during the administration period (Figure 12A). In addition, no obvious abnormality or organ damage was observed in the heart, liver, spleen, lung, or kidney (Figure 12B). These results suggest that KLA-5-FU/PTX Lps can enhance the efficacy of both chemotherapeutic drugs against TNBC without systemic side effects.

## 3. Discussion

The combination of 5-FU and PTX is commonly used in the treatment of certain human solid tumors, including breast cancer. 5-FU has a short half-life, and its metabolite fluorodeoxyuridine monophosphate prevents DNA synthesis by inhibiting thymidylate synthase, thereby suppressing tumor growth [42]. PTX has poor solubility and curbs cell division and proliferation by inhibiting tubulin depolymerization [43]. Both 5-FU and PTX distribute throughout the body, resulting in systemic toxicity. Targeting these or other drugs to TNBC is challenging because the tumors do not express ER, PR, or HER-2. Organelle-targeting drug delivery is a new strategy for tumor therapy, with the mitochondria a favored target because they play a central role in energy production and programmed cell death [44,45].

Liposomes are excellent targeted drug carriers that can be loaded with hydrophilic and lipophilic drugs, allowing for synergistic therapeutic effects. In this study, KLA-5-FU/PTX Lps were prepared using a step-by-step targeting strategy. The hydrophilic 5-FU and lipophilic PTX were co-loaded in an optimized ratio (3:2) to maximize the retention time of 5-FU and the solubility of PTX, control the release of both drugs, and shift their subcellular distribution to the mitochondria (Figure 9). In this way, the delivery system maximizes the therapeutic effect while minimizing toxicity and side effects.

Particle size, surface polarity, and surface charge all affect the efficiency of passive targeting [46,47]. We optimized these characteristics by keeping particle size within 150 nm in order to allow drug delivery to tumors via the EPR effect [48]. Second, we modified the KLA peptide using DSPE-PEG to prolong its circulation time and thereby increase drug accumulation in tumor tissues. Third, this modification substantially increased the surface polarity of the particles. The endothelial cells of tumor blood vessels have a net negative charge [49], which helps KLA peptides target tumor tissues or cells: the lysine residues on the positively charged KLA can interact with the cells via electrostatic interactions, hydrogen bonding, and hydrophobic forces, thereby facilitating uptake [38,39]. Once inside the cell, KLA is attracted to the negative charge inside the mitochondria and repulsed by the positive charge outside it, facilitating its passage inside and disrupting the mitochondrial membrane [50,51,52].

Although the combination of PTX and 5-FU has been used in the treatment of some types of solid human tumors for a long time, Johnson et al. found that free 5-FU could inhibit the cytotoxic effects of free PTX on both mitotic arrest and apoptotic cell death, probably by preventing tumor cells from entering the G_2_ m phase [53]. However, in the present study, we demonstrated that KLA-5-FU/PTX Lps had better antitumor activity than the control groups containing free drugs or unmodified liposomes, and we demonstrated this at the cellular, molecular, and whole-animal levels (Figure 6, Figure 10 and Figure 11). As mentioned above, the reason for this may be present in the differences in the subcellular distribution of both drugs. In the KLA-5-FU/PTX Lps group, the mitochondria-dominated distribution of drugs (Figure 9) replaced the pattern of the original microtubule of PTX or nucleus-dominated distribution of 5-FU individually, altering their mechanism of action and, in turn, altering their effects. In addition, these two drugs are administered at different schedules in the literature, wherein PTX is usually added prior to 5-FU [7,54]. In our study, PTX and 5-FU co-existing in liposomes still showed ideal antitumor effects; this is beneficial to reducing the number of administrations necessary, thereby improving patient compliance.

The antitumor mechanism may reflect that the KLA peptide promotes the uptake of liposomes into tumor cells and then selectively targets the mitochondria, disrupting the mitochondrial membrane. This leads to the loss of mitochondrial membrane potential, which triggers the release of cytochrome C, upregulates caspase-3 (Figure 10), and activates the apoptotic pathway in the tumor.

Chemotherapy toxicity comes mainly from the off-target distribution of drugs. The present study relied on a step-by-step targeting strategy to eliminate off-target effects and reduce systemic toxicity. Combining PTX and 5-FU allowed us to reduce the dose and thus the toxicity. Indeed, KLA-5-FU/PTX Lps showed no treatment-related systemic toxicity in cell and animal studies. Thus, using a combination of drugs and altering their subcellular distribution can provide new ideas and methods for tapping the therapeutic potential of classical drugs. In addition, the KLA peptide interacts rapidly with the cell membrane, which may help it bypass multidrug resistance and be effective against drug-resistant TNBC [55,56,57,58]. The good efficacy and safety of KLA-5-FU/PTX Lps shown here justify further pharmacokinetics and pharmacodynamics studies in comparison with injectable PTX liposomes and compound injectable polyphase 5-FU liposomes, which may bring the Lps closer to use in the clinic.

## 4. Materials and Methods

### 4.1. Materials

5-FU and PTX were purchased from Shanghai Aladdin Biochemical Technology (Shanghai, China). Soybean phosphatidylcholine (SPC), distearoyl phosphatidyl ethanolamine-polyethylene glycol (DSPE-PEG_2000_), DSPE-PEG_2000_-MAL (MAL: maleimide), and cholesterol (CHOL) were purchased from Shanghai Yuanye Biotechnology (Shanghai, China). KLA peptide was purchased from Beijing SciLight Biotechnology (Beijing, China). Chloroform and methanol were purchased from Chengdu Kelong Chemical Reagent Plant (Chengdu, China). Dulbecco’s modified Eagle’s medium (DMEM), fetal bovine serum (FBS), and phosphate-buffered saline (PBS) were obtained from Gibco (Uxbridge, UK). JC-1 apoptosis detection kit and the mitochondrial dye MitoLite Red were purchased from KeyGEN BioTECH (Nanjing, China). Annexin V-FITC/PI apoptosis detection kit, trypsin, 3-(4,5-dimethylthiazol-2-yl)-2,5-diphenyltetrazoliumbromide (MTT), coumarin-6, and dimethyl sulfoxide were obtained from Sigma-Aldrich (St. Louis, MO, USA). All other reagents were of analytical grade and were used without further purification.

### 4.2. Cell Lines and Cultures

Human umbilical vein endothelial cells (HUVECs) were purchased from Procell Life Science & Technology (Wuhan, China). MDA-MB-231, MDA-MB-453, and MDA-MB-436 human breast carcinoma cells were obtained from the State Key Laboratory of Biotherapy, Sichuan University (Chengdu, China). All the cell lines were cultured in DMEM with 10% FBS and incubated at 37 °C in a 5% CO_2_ humidified atmosphere.

### 4.3. Synthesis of DSPE-PEG-KLA

DSPE-PEG-KLA was synthesized based on a previous protocol [30]. Briefly, KLA (55.5 μmol) dissolved in methanol was added to a solution of DSPE-PEG-MAL (37 μmol) in chloroform, and the mixed solution was stirred for 24 h under nitrogen in darkness at room temperature. After confirming the complete consumption of DSPE-PEG-MAL by thin-layer chromatography, the organic solvent was removed in vacuo. The resulting white precipitate was washed three times with methanol, filtered, and analyzed with matrix-assisted laser desorption ionization time-of-flight mass spectrometry (MALDI-TOF-MS; Bruker Autoflex III; Bruker BioSciences, Billerica, MA, USA).

### 4.4. Synthesis of KLA-5-FU/PTX Lps

KLA-5-FU/PTX Lps were prepared using the thin-film dispersion method [59]. SPC (16 mg), CHOL (2 mg), DSPE-PEG_2000_ (1.5 mg), DSPE-PEG-KLA (1.5 mg), and PTX (2 mg) were dissolved in 5 mL chloroform in a round-bottom flask. This was attached to a rotary evaporator, and the organic solvent was removed by evaporating at 37 °C (60 rpm/min, 0.03 MPa), which led to the formation of the lipid film on the wall of the flask. The lipid film was dried overnight and then hydrated in 10 mL of PBS (pH 7.4) containing 5-FU (3 mg). Next, the suspension was sonicated in a bath sonicator at 37 °C for 5 min and then in a probe sonicator (JY92-IIN; Scientz Biotechnology, Ningbo, China) for 5 min (5-sec pulses at 100 W) to form KLA-5-FU/PTX Lps. Unencapsulated 5-FU and PTX were removed by refrigerated centrifugation at 10,000 rpm for 10 min, and KLA-5-FU/PTX Lps were collected by freeze-drying for 24 h. Lps lacking DSPE-PEG-KLA, 5-FU, or PTX were prepared through the same process and used as controls. All the liposomes were stored at 4 °C before use.

### 4.5. Lps Characterization

#### 4.5.1. Particle Size and Zeta Potential

The average particle size and zeta potential of Lps were measured at room temperature using a Zetasizer Nano ZS 90 (Malvern Instruments, Malvern, UK). Each preparation was tested in triplicate.

#### 4.5.2. Morphology

The morphology of the prepared KLA-5-FU/PTX Lps was analyzed using cryo-transmission electron microscopy (FEI Titan Krios Transmission Electron Microscope, Thermo Scientific, MA, USA).

#### 4.5.3. Entrapment and Drug Loading Efficiencies

The entrapment efficiency (*EE*%) and drug loading efficiency (*DL*%) of 5-FU and PTX in Lps were determined via high-performance liquid chromatography (HPLC; Agilent Technologies, Santa Clara, CA, USA) using the following formulas:

$$EE\%=\frac{\;\mathrm{weight}\;\mathrm{of}\;\mathrm{drug}\;\mathrm{in}\;\mathrm{liposomes}\;}{\mathrm{weight}\;\mathrm{of}\;\mathrm{drug}\;\mathrm{injected}}\times 100;$$

$$DL\%=\frac{\;\mathrm{weight}\;\mathrm{of}\;\mathrm{drug}\;\mathrm{in}\;\mathrm{liposomes}\;}{\mathrm{weight}\;\mathrm{of}\;\mathrm{liposomes}}\times 100.$$

### 4.6. Lps Stability In Vitro

Lps were redispersed in deionized water or DMEM (10% FBS) and stored at 4 °C for seven days, during which samples were collected at scheduled time points and analyzed for particle size and zeta potential point, as described in Section 4.5.1.

### 4.7. Hemolysis Assay of Lps

Red blood cells were collected from healthy rabbits. All animal experiments were performed in accordance with the guidelines for Care and Use of Laboratory Animals and were approved by the Experimental Animal Ethics Committee of Chengdu Medical College. The red blood cells were pelleted by centrifugation at 3000 rpm for 10 min and then suspended in normal saline to form a 2% erythrocyte suspension (*v/v*). Liposomes were mixed with normal saline and added to the erythrocyte suspension. The erythrocyte suspension was mixed with normal saline as a negative control or with deionized water as a positive control. The suspension was incubated at 37 °C for 2 h and centrifuged at 3000 rpm for 10 min. The absorbance of the supernatant was measured at 570 nm as a measure of hemolysis according to the following formula:

$$\mathrm{Hemolysis}\;(\%)=\frac{\mathrm{Abs}\;\mathrm{of}\;\mathrm{sample}\;-\;\mathrm{Abs}\;\mathrm{of}\;\mathrm{negative}\;\mathrm{control}}{\;\mathrm{Abs}\;\mathrm{of}\;\mathrm{positive}\;\mathrm{control}\;-\;\mathrm{Abs}\;\mathrm{of}\;\mathrm{negative}\;\mathrm{control}\;}\times 100.$$

### 4.8. Release of 5-FU and PTX from Lps In Vitro

The release of 5-FU and PTX from Lps was measured using a dynamic dialysis method [6]. Briefly, 5 mg of freeze-dried liposomal powder were resuspended in 1 mL of deionized water, placed into a dialysis bag with a molecular weight cut-off of 12,000 Da, and dialyzed against 100 mL of PBS (pH 5.0, 6.8, or 7.4) with shaking at 37 °C and 100 rpm. At scheduled time points, 5 mL samples were collected from the release medium and replaced with the same volume of PBS. The drug content in each sample was determined by HPLC. Each experiment was performed in triplicate.

The control free 5-FU and free PTX were prepared using the same method.

### 4.9. Synergistic Effect of 5-FU and PTX

MDA-MB-231, MDA-MB-453, and MDA-MB-436 cells were cultured in 96-well plates (5 × 10^3^ cells/well) overnight and treated with 5-FU, PTX, 5-FU/PTX (containing 5-FU concentrations of 0, 1.5, 3, 6, and 12 µg/mL and PTX concentrations of 0, 1, 2, 4, and 8 µg/mL, respectively) at 37 °C for 24 h. Cell viability was assessed using the MTT assay, and each concentration was tested in six wells. All experiments were performed in triplicate. Interaction between 5-FU and PTX was assessed using the combination index-isobologram equation based on a two-drug pharmacologic interaction model. A combination index of 1 indicates an additive effect, <1 indicates synergism, and >1 indicates antagonism. Dose–response curves and the combination index (CI) were calculated using the Compusyn Software (version 1.0, ComboSyn Inc, Paramus, NJ, USA).

### 4.10. Lps Cytotoxicity In Vitro

HUVECs, MDA-MB-231, MDA-MB-453, and MDA-MB-436 cells were cultured in 96-well plates (5 × 10^3^ cells/well) overnight and treated with 5-FU, PTX, 5-FU/PTX, 5-FU/PTX Lps, or KLA-5-FU/PTX Lps (containing 5-FU concentrations of 0, 1.5, 3, 6, and 12 µg/mL and PTX concentrations of 0, 1, 2, 4, and 8 µg/mL, respectively) at 37 °C for 24 h. Cell viability was assessed using the MTT assay, and each concentration was tested in six wells. All experiments were performed in triplicate.

### 4.11. Cell Uptake

MDA-MB-231 cells were cultured in six-well plates (2 × 10^5^ cells/well) overnight. When cells reached 70–80% confluence, the culture medium was aspirated and fresh DMEM containing free coumarin-6 (C6), C6-labeled Lps (C6 Lps), or KLA-modified C6-labeled Lps (KLA-C6 Lps) (C6: 0.1 µg/mL) was added. After incubation at 37 °C for 4 h, the cellular uptake of all preparations was observed with an inverted fluorescence microscope (IX71S1F-3, Olympus Optical, Tokyo, Japan). Further semiquantification of the cellular uptake was performed using flow cytometry. After being coincubated with free C6, C6 Lps, and KLA-C6 Lps, as described above, a certain number of cells were collected and analyzed with a flow cytometer (BD Biosciences, San Jose, CA, USA).

### 4.12. Cell Apoptosis

The apoptosis of MDA-MB-231 cells treated with different formulations was quantified using Annexin V-FITC/PI staining and flow cytometry. MDA-MB-231 cells were cultured in six-well plates (2 × 10^5^ cells/well) and incubated overnight. When cells reached 70–80% confluence, the medium was aspirated and fresh DMEM containing 5-FU, PTX, 5-FU/PTX, 5-FU/PTX Lps, or KLA-5-FU/PTX Lps (5-FU: 3 µg/mL; PTX: 2 µg/mL) was added. After incubation at 37 °C for 12 h, floating cells were collected, and adherent cells were digested with trypsin. The two cell fractions were combined and centrifuged, and the collected precipitate was incubated with 200 µL of FITC/PI buffer for 15 min on a shaker at room temperature in the dark. Cells were analyzed with a flow cytometer (BD Biosciences, San Jose, CA, USA).

### 4.13. Mitochondrial Membrane Potential

Changes in the mitochondrial membrane potential were determined using the JC-1 apoptosis detection kit following the manufacturer’s instructions. MDA-MB-231 cells were cultured in six-well plates (1.5 × 10^5^ cells/well) and incubated overnight. When the cells reached 70–80% confluence, the medium was aspirated and fresh DMEM containing 5-FU, PTX, 5-FU/PTX, 5-FU/PTX Lps, or KLA-5-FU/PTX Lps (5-FU: 3 µg/mL; PTX: 2 µg/mL) was added. After incubation at 37 °C for 12 h, the medium was aspirated, the wells were washed three times with PBS, and JC-1 staining buffer was added. After incubation at 37 °C for 20 min, the cells were washed twice with cold PBS and observed immediately with an inverted fluorescence microscope. The results were analyzed with ImageJ Software (NIH, Bethesda, MD, USA).

### 4.14. Mitochondrial Localization

MDA-MB-231 cells were cultured in six-well plates (1.5 × 10^5^ cells/well) and incubated overnight. When the cells reached 70–80% confluence, the medium was aspirated and fresh DMEM containing free C6, C6 Lps, or KLA-C6 Lps (C6: 0.1 µg/mL) was added. After incubation at 37 °C for 4 h, the medium was aspirated, and the wells were washed twice with PBS. MitoLite Red staining buffer preheated to 37 °C was then added, followed by incubation at 37 °C for 30 min. The stained cells were washed three times with PBS to remove the free dye, and the localization of the different formulations in the mitochondria was observed using a confocal laser scanning microscope (CLSM, Leica TCS SP5, Mannheim, Germany).

### 4.15. Western Blotting

MDA-MB-231 cells were cultured in six-well plates (1.5 × 10^5^ cells/well) and incubated overnight. When the cells reached 70–80% confluence, the medium was aspirated and fresh DMEM containing 5-FU, PTX, 5-FU/PTX, 5-FU/PTX Lps, or KLA-5-FU/PTX Lps (5-FU: 3 µg/mL; PTX: 2 µg/mL) was added. After incubation at 37 °C for 12 h, the medium was aspirated, and the wells were washed three times with cold PBS. After incubation with a cell lysis reagent (Beyotime Biotechnology, Shanghai, China) for 5 min, the cell suspension was aspirated and centrifuged. Proteins in the supernatant were fractionated by sodium dodecyl sulphate–polyacrylamide gel electrophoresis, transferred to polyvinylidene fluoride membranes, and then incubated with anti-caspase 3 antibody (Cell Signaling Technology, Boston, MA, USA), as described elsewhere [34]. Antibody binding was measured by enhanced chemiluminescence, and the results were analyzed using ImageJ Software.

### 4.16. In Vivo Antitumor Activity

Six-week-old female BALB/c Nude mice were purchased from Beijing Vital River Laboratory Animal Technology (Beijing, China) and maintained under standard animal house conditions.

To establish the xenograft tumor model, MDA-MB-231 cells (5 × 10^6^) suspended in PBS (0.1 mL) were subcutaneously injected into the right side of the middle part of the back of female BALB/c Nude mice. When tumors reached a uniform size of 50–100 mm^3^, mice were administered different preparations. The tumor volume was calculated as follows: tumor volume (mm^3^) = (length × width^2^)/2.

MDA-MB-231 tumor-bearing mice were then randomly divided into four groups (*n* = 6) and injected via the tail vein with saline (control), 5-FU/PTX, 5-FU/PTX Lps, or KLA-5-FU/PTX Lps (0.2 mL) at scheduled time points. The doses of 5-FU and PTX were normalized to 11.25 and 7.5 mg/kg, respectively. The tumor volume and body weight of the mice were measured every two days. At 14 days post-administration, the mice were euthanized, and the tumors were collected and weighed.

### 4.17. In Vivo Toxicity

Six-week-old normal female BALB/c Nude mice were randomly divided into four groups (*n* = 6) and injected via the tail vein with saline (control), 5-FU/PTX, 5-FU/PTX Lps, or KLA-5-FU/PTX Lps (0.2 mL) at scheduled time points. The doses of 5-FU and PTX were normalized to 11.25 and 7.5 mg/kg, respectively. The body weight of the mice was measured every two days. On day 14, the mice were euthanized, and their liver, heart, spleen, lung, and kidney were collected and fixed in 4% formaldehyde overnight. The collected tissues were then embedded in paraffin, cut using a microtome (5 µm) for hematoxylin-eosin (H&E) staining, and observed with an optical microscope (Nikon Eclipse Ci, Nikon, Tokyo, Japan).

### 4.18. Statistical Analysis

Statistical analysis was performed with SPSS 13.0 (IBM, Chicago, IL, USA). All the data are expressed as the mean ± standard deviation (SD). A one-way analysis of variance (ANOVA) was used in the study to determine significant differences between pairs of two groups. Differences associated with *p* < 0.05 were considered statistically significant.

## 5. Conclusions

We developed a KLA-modified liposome-based nanosystem for the co-delivery of 5-FU and PTX into the mitochondria of TNBC cells. The developed formulation may improve the therapeutic efficacy of both drugs while reducing systemic toxicity and improving safety. KLA-5-FU/PTX Lps should be able to accommodate drugs with different physicochemical properties, so it may be useful for combination therapy against various cancers.

## Figures and Tables

**Table 1:** Physical properties of blank, 5-FU/PTX, and KLA-5-FU/PTX Lps.

Liposomes	Size/nm	PDI	Zeta Potential/mV	*EE*%/*DL*% (5-FU)	*EE*%/*DL*% (PTX)
Blank Lps	123.68 ± 4.63	0.243 ± 0.015	−17.87 ± 2.56	-	-
5-FU/PTX Lps	125.27 ± 2.08	0.154 ± 0.009	−16.51 ± 3.62	84.75 ± 1.24/ 8.61 ± 1.68	82.36 ± 3.82/ 6.92 ± 1.54
KLA-5-FU/PTX Lps	130.56 ± 3.14	0.238 ± 0.011	16.83 ± 1.95	82.81 ± 2.16/ 7.99 ± 1.36	80.47 ± 2.39/ 6.53 ± 1.03

All preparations were tested in triplicate. 5-FU, 5-fluorouracil; PTX, paclitaxel; Lps, liposomes; PDI, polydispersity index; *EE*, entrapment efficiency; *DL*, drug loading efficiency.

**Table 2:** IC_50_ values of different drug groups against different TNBC cells.

Cell lines	IC_50_ (µg/mL)				
	5-FU	PTX	5-FU/PTX	5-FU/PTX Lps	KLA-5-FU/PTX Lps
MDA-MB-231	7.66 ± 0.54	5.17 ± 0.51	2.97 ± 0.47	1.62 ± 0.23	0.85 ± 0.07
MDA-MB-453	9.54 ± 0.76	8.10 ± 0.74	4.44 ± 0.66	2.47 ± 0.12	2.10 ± 0.04
MDA-MB-436	6.24 ± 0.69	5.35 ± 0.72	2.14 ± 0.18	1.64 ± 0.15	1.24 ± 0.06

All preparations were tested in triplicate. IC_50_, half-maximal inhibitory concentration. 5-FU, 5-fluorouracil; PTX, paclitaxel; 5-FU/PTX, mechanically mixed free 5-FU and free PTX; 5-FU/PTX Lps, unmodified liposomes co-loaded with 5-FU and PTX; KLA-5-FU/PTX Lps, KLA-modified liposomes co-loaded with 5-FU and PTX.

## Data Availability

Data are contained within the article.
